# Oral vaccination of fish against vibriosis using spore-display technology

**DOI:** 10.3389/fimmu.2022.1012301

**Published:** 2022-10-13

**Authors:** Gabriela Gonçalves, Rafaela A. Santos, Filipe Coutinho, Neide Pedrosa, Maria Curado, Marina Machado, Benjamin Costas, Lourenço Bonneville, Mónica Serrano, António Paulo Carvalho, Patricia Díaz-Rosales, Aires Oliva-Teles, Ana Couto, Cláudia R. Serra

**Affiliations:** ^1^ Departamento de Biologia, Faculdade de Ciências, Universidade do Porto, Porto, Portugal; ^2^ Centro Interdisciplinar de Investigação Marinha e Ambiental, Universidade do Porto (CIMAR/CIIMAR), Matosinhos, Portugal; ^3^ Faculdade de Ciências e Tecnologia, Universidade NOVA de Lisboa, Caparica, Portugal; ^4^ Instituto de Ciências Biomédicas Abel Salazar, Universidade do Porto, Porto, Portugal; ^5^ Laboratory of Microbial Development, Instituto de Tecnologia Química e Biológica António Xavier (ITQB-NOVA), Oeiras, Portugal; ^6^ Fish Immunology and Pathology Group, Animal Health Research Centre (CISA, INIA-CSIC), Madrid, Spain

**Keywords:** oral vaccines, aquaculture, bacterial spores, *Bacillus*, zebrafish, european seabass

## Abstract

Oral vaccines are highly demanded by the aquaculture sector, to allow mass delivery of antigens without using the expensive and labor-intensive injectable vaccines. These later require individual handling of fish, provoking stress-related mortalities.

One possible strategy to create injection-free vaccine delivery vehicles is the use of bacterial spores, extremely resistant structures with wide biotechnological applications, including as probiotics, display systems, or adjuvants. Bacterial spores, in particular those of *Bacillus subtilis*, have been shown to behave as mucosal vaccine adjuvants in mice models. However, such technology has not been extensively explored against fish bacterial disease.

In this study, we used a laboratory strain of *B. subtilis*, for which a variety of genetic manipulation tools are available, to display at its spores surface either a *Vibrio* antigenic protein, OmpK, or the green fluorescence protein, GFP. When previously vaccinated by immersion with the OmpK- carrying spores, zebrafish survival upon a bacterial challenge with *V. anguillarum* and *V. parahaemolyticus*, increased up to 50 - 90% depending on the pathogen targeted. Further, we were able to detect anti-GFP-antibodies in the serum of European seabass juveniles fed diets containing the GFP-carrying spores and anti-*V. anguillarum* antibodies in the serum of European seabass juveniles fed the OmpK-carrying spores containing diet. More important, seabass survival was increased from 60 to 86% when previously orally vaccinated with in-feed OmpK- carrying spores. Our results indicate that *B. subtilis* spores can effectively be used as antigen-carriers for oral vaccine delivery in fish.

## Introduction

Aquaculture is the fastest-growing food-producing sector. According to the Food and Agriculture Organization of the United Nations (FAO), bacterial diseases are a major constraint to the economic and sustainable development of aquaculture, with a global negative impact of tens of billions of US$ in the last 20 years (1, 2). Besides massive animal losses, bacterial disease outbreaks are associated with the misuse of antibiotics, creating selective pressure for the emergence of drug-resistant bacteria, rendering antibiotic treatments ineffective, and leaving residues in aquaculture products for human consumption, both are serious threats to public health (3). Also, the zoonotic potential of some fish pathogens has previously been described (4). In a post-antibiotic era, where a decreasing efficacy of antimicrobials might turn minor infections into a global problem, it is urgent to find alternatives that assure advanced and integrated health care for humans, animals, and the environment.

Disease prevention by vaccination is, on economic, environmental, and ethical grounds perceived as the most appropriate method for pathogen control in the aquaculture sector. Most licensed fish vaccines are based on inactivated microorganisms, formulated with adjuvants, and delivered through injection or, to a less extent, by immersion (5, 6). Injectable vaccines are labor-intensive, expensive, require individual handling of fish, provoking stress-related immunosuppression, and handling mortalities (5, 6). Also, vaccination by injection is not feasible for early fish stages, even though it is precisely at these early stages when vaccination is more often needed. Additionally, efficient vaccines against a wide range of aquaculture diseases still have to be developed (5–7).

One of the most problematic bacterial diseases in aquaculture is Vibriosis, an haemorrhagic septicaemia caused by different species of the Gram-negative genus *Vibrio* (8–10), affecting not only fish but also shrimps and bivalves (8–10). *Vibrio* spp. can also affect humans with the American Centre for Diseases Control (CDC) estimating that vibriosis causes 80000 illnesses each year in the United States, with around 52000 being the result of eating contaminated food. *V. anguillarum*, the etiological agent of classical vibriosis in warm- and cold-water fish species, is particularly problematic, leading to high mortalities and economic losses in aquaculture (9, 10). Although commercial vaccines are currently available for aquaculture vibriosis (e.g., *Lipogen Forte*), these are mainly injectable vaccines, with the disadvantages highlighted above, and based on inactivated pathogens.

Present vaccine trend developments focus on alternative methods for mass delivery of antigens, including oral and immersion vaccination (5, 6, 11). Oral vaccine administration incorporated in feed seems to be the preferable method, because it is more versatile for immunization against a wider range of pathogens, reduces fish handling stress and costs to the minimum, and is feasible for larvae and juveniles vaccination (5, 6, 11). Despite their high demand by the aquaculture sector, most previous attempts to obtain effective oral vaccines have failed (5, 6, 11). The few available are commercialized as boosts to previous injection vaccination strategies and are not intended to be used on their own (5, 6, 11, 12). This is mainly because i) many aspects of fish immunology are still unknown; ii) mucosal vaccination is still in the early steps of development, including for humans and terrestrial animals and, iii) oral vaccine success in fish is highly dependent on the correct antigen-adjuvant combination, with previous attempts failing to elicit an effective immune response and protection upon a bacterial challenge (5, 6, 11–14).

One possible strategy to create a novel oral vaccine delivery platform is the use of bacterial spores, extremely resistant structures with wide biotechnological applications, including probiotics and display systems (15–17). Bacterial spores, in particular those of *Bacillus subtilis*, have been shown to behave as mucosal vaccine adjuvants in mice models (18, 19), promoting the increase of antibody responses after co-administration with antigens either mixed or adsorbed on the spore surface. Although several successful examples exist of the use of *B. subtilis* spores as vaccine delivery vehicles against fish viral (20–28) and parasitic diseases (29–31), to date, such technology has only been applied once in the prevention of bacterial diseases of aquatic animals, namely against the Gram-positive *Streptococcus agalactiae* (32). Moreover, all studies published so far have targeted only diseases of crustaceans (20–24) or freshwater fish species (25–31), namely tilapia or grass carp. To fulfil these gaps, in this study, we developed a spore-based vaccine platform (Vaccine) displaying an antigen of *Vibrio* spp., causing one of the most problematic bacterial diseases affecting aquaculture (4, 10, 33) and, evaluated its effectiveness in protecting the model zebrafish (*Danio rerio*) and the marine aquaculture European seabass (*Dicentrarchus labrax*) against problematic *Vibrio* pathogens.

## Methods

### Bacterial strains, culture conditions, and general methods

Bacterial strains used in this study are listed in **Table 1**. Luria-Bertani (LB; Fisher BioReagents) medium was used for the routine growth and transformations of *Bacillus subtilis* and *Escherichia coli* strains. Brain Heart Infusion (BHI; Becton Dickinson) medium was used for the growth of *V. vulnificus*, *V. anguillarum*, and *V. parahaemolyticus*. Difco sporulation medium (DSM; Becton Dickinson), was used to induce sporulation of *B. subtilis*. Bacterial growth was performed at 37°C (*B. subtilis* and *E. coli*) or 28°C (*Vibrio.* spp) overnight (with agitation at 140 rpm when cultured in liquid medium). When appropriate, chloramphenicol (5 μg mL^-1^) and ampicillin (100 μg mL^-1^) were used for the growth of *B. subtilis* and *E. coli* strains, respectively.

**Table 1:** Bacterial strains and plasmids used in this work.

Name	Relevant genotype/phenotype ^a^	Source, Reference or construction ^b^
**Bacterial strain**		
*E. coli* DH5α	fhuA2Δ(argF-lacZ)U169 phoA glnV44 Φ80 Δ(lacZ)M15 gyrA96 recA1 relA1 endA1 thi-1 hsdR17	Commercial strain (Nzytech MB004)
*B. subtilis* 168	*trpC2*	A.O. Henriques
*Vibrio vulnificus* LMG 13545	Type Strain (ATCC 27562)	BCCM/LMG
*Vibrio anguillarum* DSM 21597	Type Strain (ATCC 19264)	DSMZ
*Vibrio parahaemolyticus* LMG 2850	Type Strain (ATCC 17802)	BCCM/LMG
CRS218	Δ*amyE*::P*_cotYZ_-cotY-6His-gfp*, Cm^R^	*B. subtilis* 168 x pGG7
CRS219	Δ*amyE*::P*_cotYZ_-6His-gfp-cotY*, Cm^R^	*B. subtilis* 168 x pGG8
CRS220	Δ*amyE*::P*_cotYZ_-cotY-6His-ompK*, Cm^R^	*B. subtilis* 168 x pGG9
CRS221	Δ*amyE*::P*_cotYZ_-6His-ompK-cotY*, Cm^R^	*B. subtilis* 168 x pGG10
CRS239	amyE::P*_veg_*-*mCherry*, Cm^R^	*B. subtilis* 168 x pCS114
**Plasmid**		
pDG364	*bla amyE3’ cat amyE5’*	(34)
pMS157	*bla km gfp*	(35)
p1CSV-CotY-C	*bla amyE3´ P_cotYZ_-cotY-rfp cat amyE5´*	(36)
p1CSV-CotY-N	*bla amyE3´ P_cotYZ_-rfp-cotY cat amyE5´*	(36)
pSB1C3-mCherry	*bla cat mCherry*	(36)
pGG7	*bla amyE3´ P_cotYZ_-cotY-6His-gfp cat amyE5´*	This study
pGG8	*bla amyE3´ P_cotYZ_-6His-gfp-cotY cat amyE5´*	This study
pGG9	*bla amyE3´ P_cotYZ_-cotY-6His-ompK cat amyE5´*	This study
pGG10	*bla amyE3´ P_cotYZ_-6His-ompK-cotY cat amyE5´*	This study
pCS114	*bla amyE3’ P_veg_-mCherry cat amyE5’*	This study

a Antibiotic resistance indicated: Cm – chloramphenicol.

b Bacterial strains were obtained from bacterial collections (BCCM/LMG, Belgian Coordinated Collections of Microorganisms, Laboratory of Microbiology, Department of Biochemistry and Microbiology, Faculty of Sciences of Ghent University, Ghent, Belgium; DSMZ, DSM Collection, German Collection of Microorganisms and Cell Cultures, Braunschweig, Germany; from our laboratory stocks (NUTRIMU collection) or kindly supplied by A. O. Henriques (Instituto de Tecnologia Química e Biológica António Xavier, Universidade Nova de Lisboa, Portugal).

Genomic DNA from *B. subtilis* and *V. vulnificus* (**Table 1**) was extracted, using ZymoBIOMICS DNA Miniprep kit (Zymoresearch), from 2 mL LB or BHI cell suspensions, respectively, previously grown overnight at 37°C or 28°C respectively, from a single fresh colony. All PCR reactions were performed with Phusion™ High-Fidelity DNA Polymerase (Thermo Scientific^™^) according to the manufacturer’s instructions.

### Construction of *B. subtilis* strains carrying OmpK and GFP fusions to CotY

An N-terminal 6-Histidines-Tag was placed by PCR on the target proteins, OmpK and GFP, before fusing to CotY, an abundant protein present in the crust of the spore. A 787 bp DNA fragment containing the coding sequence of *ompK* but excluding its first 72 nucleotides (coding for a signal peptide), was PCR amplified from *V. vulnificus* chromosomal DNA using oligonucleotide primers 6His-ompK-F and ompK-SpoVec-R (**Table 2**). To obtain the GFP fusion to CotY, plasmid pMS157 (**Table 1**) was used as the template for PCR amplification of *gfp* with oligonucleotides 6His-gfp-F and gfp-SpoVec-R (**Table 2**), resulting in a 769 bp fragment carrying the 6-his tagged-GFP. Following purification of the DNA fragments, a second PCR with oligonucleotide primers 6His-SpoVec-F and ompK-SpoVec-R (in the case of OmpK) or gfp-SpoVec-R (for GFP) (**Table 2**) originated an 827 bp or an 809 bp product, respectively.

**Table 2:** List of oligonucleotide primers used in this study. Native or introduced restriction sites are indicated in different colors (NgoMIV, Xbal, AgeI, SpeI, HindIII, EcoRI).

Primer name	Sequence (5’-3’)
6His-SpoVec-F	GATCGAATTCGCGGCCGCTTCTAGAAAGGAGGTGGCCGGCATGCATCACCATCACCATCAC
6His-gfp-F	ATGCATCACCATCACCATCACATGAGTAAAGGAGAAGAACTTTTC
gfp-SpoVec-R	AGCTCTGCAGCGGCCGCTACTAGTATTAACCGGTTTTGTATAGTTCATCCATGCCATG
6His-ompK-F	ATGCATCACCATCACCATCACGACGGCGATATCCACAAAAACGAT
ompK-SpoVec-R	AGCTCTGCAGCGGCCGCTACTAGTATTAACCGGTCTTGTAAGTTACTGCGACGTAGTG
Pveg-F (HindIII)	CCCAAGCTTAATTTTGTCAAAATAATTTTATTGACAACG
Pveg-RBS-SpoVG-R	TTCACCACCTTTCTCTAGTAACATTTATTGTACAACACGA
mTagBFP-RBS-SpoVG-F	GAAAGGTGGTGAATACTAGATGGCCGGCGTTAGCAAAGGCGAAG
mCherrymTagBFP-R	CCGGAATTCTTAACCGGTTTTATACAGTTCATCCATTCC
amyE-F	CGGTTTGAAAGGAGGAAGCGGAAGAATG
amyE-R	CAAAGCCAGGCTGATTCTGACCGGGCAG

For the N-terminal variants, each DNA fragment previously digested with XbaI (Anza^™^ Invitrogen) and AgeI (Thermo Scientific) was ligated to p1CSV-CotY-N (**Table 1**) cleaved with XbaI and NgoMIV (New England BioLabs), yielding pGG7 and pGG9 (**Table 1**). For the C-terminal variants, each DNA fragment digested with NgoMIV and SpeI (Thermo Scientific) was inserted into p1CSV-CotY-C (**Table 1**) cleaved with SpeI and AgeI, yielding pGG8 and pGG10 (**Table 1**). Transformation of *B. subtilis* 168 competent cells prepared following established procedures (34) with ScaI linearized pGG7, pGG8, pGG9, and pGG10 originated the chloramphenicol resistant (CmR) strains CRS218, CRS219, CRS220, and CRS221, respectively (**Table 1**).

### Construction of *B. subtilis* strain carrying a *Pveg-mCherry* transcriptional fusion

To fuse the constitutively active promoter P*veg* to *mCherry*, a 126 bp DNA fragment comprising the regulatory region of the *veg* gene was first amplified by PCR from chromosomal DNA of *B. subtilis* 168 using primers Pveg-F-HindIII and Pveg-RBSSpoVG-R (which contains the RBS of *spoVG* gene) (**Table 2**). Next, the *mCherry* coding region (plus 19 bases upstream of its start codon and 9 bases downstream of its stop codon) was PCR amplified from pSB1C3-mCherry (ECE757) (37) (**Table 1**) with primers mTagBFP-RBS-SpoVG-F (which contains the RBS of *spoVG* gene, as above) and mCherrymTagBFP-R (**Table 2**), introducing an EcoRI restriction site at the 3’end of the PCR product. An overlapping PCR was then performed between both P*veg* and *mCherry* fragments, using primers Pveg-F-HindIII and mCherrymTagBFP-R (**Table 2**). The purified PCR product was then inserted into plasmid vector pDG364 (**Table 1**) after digestion with HindIII and EcoRI, originating pCS114 (**Table 1**). Transformation of *B. subtilis* 168 competent cells prepared as described above originated the chloramphenicol resistant (Cm^R^) strain CRS239 (**Table 1**).

### Spores purification and immunodetection of spores’ displayed GFP and OmpK

Spores of wild type (WT) *B. subtilis* 168 and its congenic derivatives CRS218, CRS219, CRS220 and CRS221, were obtained after sporulation induction by nutrient exhaustion in DSM (38) and purified as previously described (36).

Spore suspensions were quantified at 580 nm, mixed with 2X Loading Buffer (10% glycerol, 10% 2-mercaptoethanol, 100 mM Dithiothreitol (DTT), 4% SDS, 0.05% bromophenol blue, 0.125 M Tris), boiled for 8 min, and spore coat proteins resolved by SDS-PAGE on a 15% acrylamide gel before staining with Coomassie Brilliant Blue R-250. For the western blot analysis, the acrylamide resolved proteins were electrotransferred to a 0.2 µm nitrocellulose membrane (BioRad) in cold transfer buffer (0.192 M glycine, 0.025 M Tris, 10% ethanol, pH=8.3). Following a 30 min blocking step with PBS-10% (w/v) low fat milk, the membrane was washed three times with 1XPBS-0.1% (v/v) Tween and incubated with 1:1000 6His-Tag monoclonal antibody (HIS6.H8; Invitrogen) overnight at 4°C. The next day, the membrane was again washed and incubated with 1:10000 goat anti-mouse IgG (H+L) HRP conjugated (Invitrogen) for 30 min, before signal detection using the Clarity Western ECL Substrate ECL detection solution (Bio-Rad) following the manufacturer instructions. Both Coomassie-stained polyacrylamide gel and western blot were visualized in a ChemiDoc XRS Gel Imaging System (Bio-Rad) and analyzed with the Image Lab Software (Bio-Rad).

### Phase-contrast and fluorescence microscopy

**Figure 1:**
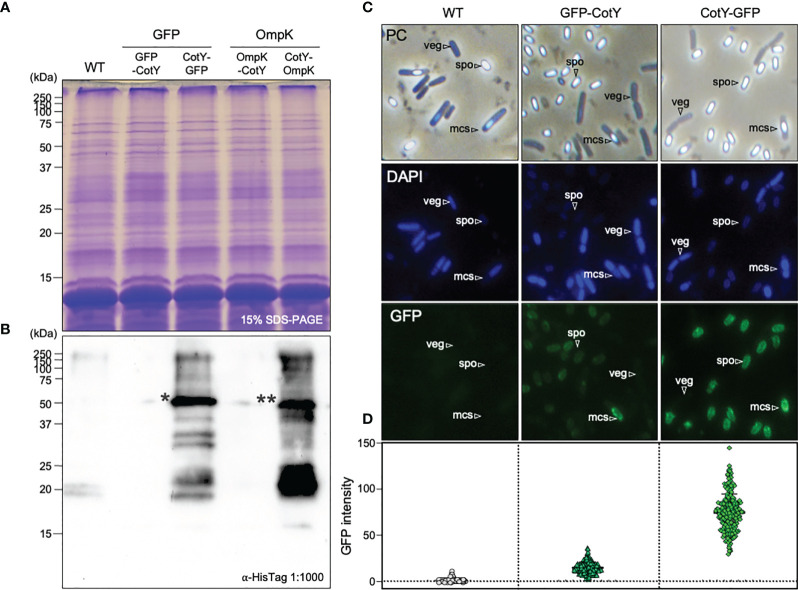
Detection of GFP and OmpK proteins at the surface of *B subtilis* spores. **(A)** Spore proteins from *B subtilis* 168 (WT) and its congenic derivatives bearing the N- and C-terminal fusions of GFP (CRS219, CRS218) and OmpK (CRS221 and CRS222) to CotY, were separated in a 15% acrylamide gel, all revealing a similar pattern. **(B)** Western blot analysis using an anti-HisTag primary antibody (1:1000) detected two bands (indicated asterisks) of approximately 46KDa equivalent to the sum of the MW of CotY (18KDa) and H6-GFP (28KDa) indicated as * or H6-OmpK (28 KDa) indicated as ** in the C-terminal clones. The molecular weight marker (MWM) sizes are indicated in KDa. **(C)** Phase-contrast (PC) and fluorescence microscopy images of 24h cultures of *B subtilis* 168, CRS218 and CRS219 grown in DSM at 37°C and 150 rpm, stained with DAPI for detecting the nucleoid. Vegetative cells (veg), sporulating cells (mcs) and free mature spores (spo) are indicated. **(D)** GFP signal intensity quantification in 200 cells per strain with ImageJ software (https://imagej.nih.gov/ij).

Sporulating cultures of strains *B. subtilis* 168, CRS218, CRS219, and CRS239, grown for 24 h (4 h in the case of CRS239) after the onset of sporulation or T0 in DSM, were observed by phase-contrast and fluorescence microscopy using a Nikon Eclipse Ci-L microscope with a CoolLED’s pE 300lite illumination system equipped with a 100x oil objective. Samples preparation was done as previously described (38). Each culture was observed under the same filter conditions: exposure of 50 mms for visualization of GFP (FITC filter; green fluorescence) and mCherry (Texas Red filter, red fluorescence), and 20 mms for visualization of DAPI (blue fluorescence). Images were captured using a DS-Ri2 Nikon Camera and processed with NIS-Elements BR v. 4.60.00 software (Nikon Corporation). Quantification of GFP intensity of fluorescence (**Figure 1D**) was done in 200 cells per strain with the ImageJ software (https://imagej.nih.gov/ij).

### Animals and *in vivo* experimental conditions

Zebrafish larvae were produced by pair-wise mating of wild-type adults, collected, and raised at 28°C with natural photoperiod. From 5 days-post-fertilization (dpf) larvae were fed twice a day. At the end of each assay, larvae were euthanized with an overdose of tricaine methanesulfonate (MS-222, 300 mg L^-1^).

A preliminary test (data not shown) was carried out to assess the toxicity potential of *B. subtilis* 168 spores, as follows: at 3 dpf, zebrafish larvae were distributed into 6-well plates containing 10 larvae per well in 5 mL of Egg water (26.4 mg L^-1^ Instant Ocean^®^ Salt). Serial dilutions of purified spores’ suspension were carried out in different wells, from 10^4^ CFU mL^-1^ to 10^8^ CFU mL^-1^. A negative control without spores’ suspension was also included. Larvae survival was monitored for 72 h.

Additionally, 6 dpf zebrafish larvae (previously exposed to 75 µM PTU (Phenylthiourea) to inhibit embryos’ pigmentation) (39) were immersed with 10^8^ CFU mL^-1^ of strain CRS239 (Pveg-mCherry), and incubated for 24 h in the same conditions before observation by phase-contrast and fluorescence microscopy, as described above with adjustment of the objective magnification.

### Immunization by immersion and challenge of zebrafish

To evaluate the protective effect of OmpK-carrying spores, 4 dpf larvae were distributed into 6-well plates containing 10 larvae per well in 5 mL of Egg water to acclimatize to the experimental conditions. At 6 dpf, larvae were treated for 2 h, [following previously established protocols (40)], with spores suspensions containing 1x10^8^ CFU mL^-1^ of each recombinant strain (CRS220, CotY-H6-OmpK and CRS221, H6-OmpK-CotY), or the parental WT *B. subtilis* 168 strain. At 9 dpf, larvae were challenged by immersion during 12h with 3×10^8^ CFUs mL^-1^ of *V. anguillarum* or 1×10^8^ CFUs mL^-1^ of *V. parahaemolyticus* (following established models of infection previously developed by us, unpublished data). Cumulative mortalities were registered during 24h, and dead larvae were removed and safely discarded. Control groups included: (i) non-vaccinated larvae challenged with *V. anguillarum* or *V. parahaemolyticus*; (ii) non-inoculated larvae and (iii) larvae inoculated with 1X phosphate-buffered saline (PBS), the diluent of each bacterial inoculum. The experiment was independently carried out 3 times.

### European seabass oral vaccination with GFP and OmpK-carrying spores and challenged with *V. anguillarum*

The trial was performed at the experimental facilities of CIIMAR, Porto, Portugal. European seabass (*Dicentrarchus labrax*) juveniles were obtained from a commercial fish farm (Maresa S.A., Ayamonte, Huelva, Spain), and submitted to a quarantine period of 15 days before being transferred to an experimental recirculating water system (RAS) equipped with 9 cylindrical fiberglass tanks of 300 L water capacity and thermo-regulated to 22.0 ± 1.0°C. Tanks were supplied with a continuous flow of filtered seawater (Salinity: 34.0 ± 1.0 g L^-1^; Oxygen 7.5 mg L^-1^; ${\text{NH}}_{4}^{+}$ ≤ 0.05 mg L^-1^; ${\text{NO}}_{2}^{-}$ ≤ 0.5 mg L^-1^). After 15 days of adaptation to the experimental conditions, 40 European seabass with an initial mean body weight of 26.5 g were randomly distributed to each tank (making triplicate groups) and to the experimental diets as follows: CTR (or non-vaccinated group): fish fed the non-supplemented commercial diet; SPO: fish fed the commercial diet supplemented with 1x10^9^ CFU Kg^-1^ of CotY-OmpK carrying spores (strain CRS220, **Table 1**); GFP: fish fed the commercial diet supplemented with 1x10^9^ CFU Kg^-1^ of CotY-GFP carrying spores (strain CRS218, **Table 1**). Lyophilized spores were incorporated in finely grounded commercial diet, well mixed, and dry pelleted in a laboratory pellet mill (California Pellet Mill, CPM, Crawfordsville, IN, USA) through a 2 mm die. The pellets were then dried in an oven at 40°C for 24 h and stored in airtight bags until used. Fish were fed daily by hand, until apparent visual satiation, for 30 days. Blood of 4 fish/tank (12 fish per treatment) was collected on day 30 for serum antibody analysis.

Then, the OmpK-vaccinated and the non-vaccinated fish were submitted to a bacterial challenge by intraperitoneal (i.p.) injection with 100 µl of *V. anguillarum* DSMZ 21597 (1x10^7^ CFU mL^-1^; previously established target LD50) (challenged fish) or with 100 µl of 1xPBS (non-challenged fish). The rearing system was monitored twice a day and water quality parameters were maintained in the same conditions described above. Mortalities were recorded for 7 days, with dead and moribund fish daily removed or sacrificed and safely discarded. To ensure that fish mortalities were related to the *V. anguillarum* infection, the head kidney of 3 moribund fish was harvested and plated in Thiosulfate-citrate-bile salts-sucrose agar (TCBS; Merck), Marine agar (MA; Becton Dickinson), and Tryptic soy agar (TSA; Becton Dickinson). Pathogen confirmation was accessed through PCR amplification of the 16S rRNA gene using universal primers 16S-27F and 16S-1492R. At the end of the challenge, the remaining fish were euthanized with an overdose (1mL L^-1^) of anesthetic (Ethylene glycol monophenyl ether; Merck) and safely discarded.

### Dot blot and ELISA analysis of European seabass serum

Dot blot analysis was done with 50 ng of GFP protein purified as previously described (41) against 10 µl of immobilized undiluted fish serum (2 pools of 6 fish, 2 fish from each tank) of either vaccinated (fish fed GFP-carrying spores supplemented diet) or non-vaccinated (control fish fed un-supplemented diet) animals, or Bovine Serum Albumin (BSA). An anti-GFP-specific primary antibody (42) was used followed by incubation with a Goat anti-Rabbit IgG (H+L) HRP-conjugated secondary antibody (Invitrogen).

ELISA analysis was done in 96-well microtiter plates (Nunc Flat bottom *MaxiSorp™*) coated with 100 µl of a freshly prepared *Vibrio anguillarum* “antigen solution” (consisting of a cell pellet from an overnight grown culture, with each well containing approximately 4.8 x 10^7^ CFU mL^-1^) and incubated at room temperature for 2 h. The wells were then washed with 200 µl of 1X PBS-0.1% Tween and unspecific binding was blocked with 200 µl of 1X PBS-10% low-fat milk. After 2 h, the wells were washed and then coated with 100 µl of fish serum diluted at 1:6 in 1X PBS or with BSA (20 µg mL^-1^), and incubated overnight at 4°C. Wells coated with 1X PBS (serum diluent) were also included. The next day, wells were washed and coated with 100 µl of Anti-European seabass IgM monoclonal antibody (Aquatic Diagnostics Ltd.), diluted at 1:33 in 1X PBS, and incubated at room temperature for 1 hour. After washing, wells were incubated for 1 hour with 100 µl of Goat anti-Mouse IgG (H+L) HRP-conjugated secondary antibody (Invitrogen) diluted at 1:10000 in 1X PBS, which was then replaced by 100 µl of TMB (3,3’,5,5’-tetramethylbenzidine) for twenty minutes before measuring antibody binding. The reaction was stopped by adding 100 µl of 2M H_2_SO_4_ and read immediately at an absorbance of 450nm.

### Data analysis

Data analysis was done using the SPSS 26.0 software package for Windows or GraphPad Prism 8 software. Survival was analyzed using Kaplan–Meier and group differences were evaluated by the log-rank analysis. A *p*<0.05 was considered significant. Dunnett’s test was performed for comparisons between treatments and the control.

## Results

### CotY is an efficient carrier for protein display at the surface of *B. subtilis* spores when fused C-terminally but not when at the N-terminus

The crust protein CotY was chosen as the carrier for spore surface display of both GFP and OmpK proteins. While the full sequence of GFP was fused to CotY, in the case of the OmpK outer-membrane protein, the first 72 nucleotides of its coding sequence were excluded to eliminate the signal peptide and to initiate at a conserved DNA region among several *Vibrio* spp. (**Supplementary Figure 1**).

Fusions to CotY were done at both the N and C-terminus, as the success of protein display at the surface of the spores is protein-specific and highly variable whether it is C- or N-terminally fused. Since CotY is a cysteine rich protein that forms very stable complexes, that are difficult to solubilize (43, 44), CotY is not easily identified in a normal SDS-PAGE stained with coomassie, and antibodies are usually used for the detection of recombinant proteins (45–49). For that purpose, one tag of 6 histidines was placed at the N-terminal ends of both GFP and OmpK, to be able to detect the recombinant proteins using a commercial antibody with established specificity and efficacy, without the need to produce anti-OmpK antibodies. As a negative control, we use the WT strain which does not carry the his-tag.

Proteins from purified spores of *B. subtilis* 168 (WT, without fusion proteins) and its congenic derivatives bearing C- or N-terminal fusions of GFP (CRS218, CRS219) or OmpK (CRS220, CRS221) to the crust protein CotY had similar patterns when separated in a 15% SDS-PAGE (**Figure 1A**), indicating no major visible alterations on the spore protein profile, meaning that the fusion protein does not interfere with the normal assembly of other proteins into the spore. Subsequent western blot analysis with an anti-HisTag antibody recognized bands of the expected size (~46 kDa) in the case of C-terminal fusions CotY-H6-GFP (46.02 kDa) and CotY-H6-OmpK (46.47 kDa) but not on the corresponding N-terminal versions (**Figure 1B**). As expected, no bands were detected in the control WT. Sequencing of plasmids pGG7, pGG8, pGG9, and pGG10 (**Table 1**) and of PCR amplification of the *amyE* region with primers AmyE-F and AmyE-R (**Table 2**) from genomic DNA of strains CRS18, CRS219, CRS220, and CRS221 (**Table 1**) revealed a correct sequence assembly (data not shown).

Microscope observation showed that spores of strains bearing both C-terminal and N-terminal fusion of CotY to GFP (CRS218 and CRS219 respectively) exhibited green fluorescence (**Figure 1C**). This not only indicates a successful display of GFP at the spores’ surface, confirming the western-blot results in the case of the C-terminal version CRS218 but also the display of a functional GFP protein. Moreover, we could confirm that both strains produced mature spores, i.e. phase-bright spores under phase-contrast microscopy, indicative of heat resistance, and impermeable to DAPI DNA staining. The spores’ coat avoids the entry of this agent, as opposed to the blue coloration observed in vegetative and sporulating cells.

Spores harboring the C-terminal fusion CotY-H6-GFP (the only one detected by western blot analysis) exhibited higher intensity of fluorescence as if the display was more efficient in this version (**Figure 1C**). Accordingly, following quantification, the average GFP expression level at the surface of the recombinant spores was much higher in the C-terminal version (**Figure 1D**).

All recombinant strains exhibited a pattern of bacterial growth and sporulation rate similar to the parental strain *B. subtilis* 168, initiating the process of sporulation approximately 4 h after the initial inoculation, which corresponds to the beginning of the stationary phase (50) Spores resistance to heat and lysozyme of the recombinant strains was also unaffected (data not shown).

### OmpK-carrying spores protect zebrafish larvae from infection with *V. anguillarum* and *V. parahaemolyticus*

**Figure 2:**
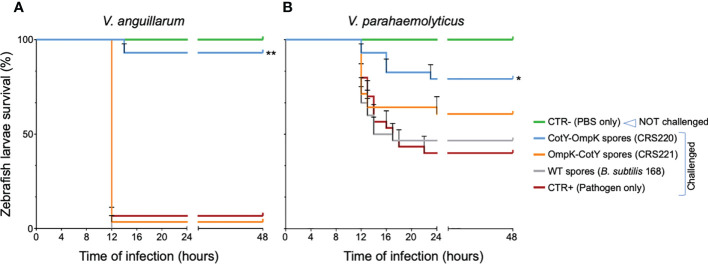
Zebrafish larvae survival to *V. anguillarum* and *V. parahaemolyticus* infection, after exposure by immersion to OmpK carrying spores. Kaplan-Meyer survival curves of zebrafish larvae upon challenge with *V. anguillarum* **(A)** or *V. parahaemolyticus* **(B)**, three days after being vaccinated with spores from *B subtilis* 168 (WT) or its congenic derivatives bearing the OmpK antigen fused to CotY, namely strains CRS220 (CotY-H6-OmpK) and CRS221 (H6-OmpK-CotY). Non-vaccinated larvae (CTR+, pathogen only) and non-challenged larvae (CTR-, PBS only) were used as positive and negative control groups, respectively. Larvae were monitored for 48 hours. Data are represented as mean ± standard deviation of three independent biological experiments. Significant differences are highlighted by asterisks: * (*p*<0.05) or ** (*p*<0.001).

Challenging zebrafish larvae with *V. anguillarum* was more deadly than challenging with *V. parahaemolyticus*, revealing the different pathogenic characteristic of both strains. Infection with *V. anguillarum* caused almost total mortality within 12 h, while when exposed to *V. parahaemolyticus* 40% of larvae survived after 48 h (**Figure 2**). When previously treated (vaccinated) by immersion with spores from the WT strain *B. subtilis* 168 and from its congenic derivative CRS221 (H6-OmpK-CotY), no significant improvement in larvae survival was observed. On the contrary, vaccination with spores exhibiting the fusion CotY-H6-OmpK (CRS220) induced a significant protective effect against both pathogens (**Figure 2**): larvae survival increased from 40 to 80% upon challenge with *V. parahaemolyticus* (*p*<0.05) and from 5 to 90% when challenged with *V. anguillarum* (*p*<0.001).

**Figure 3:**
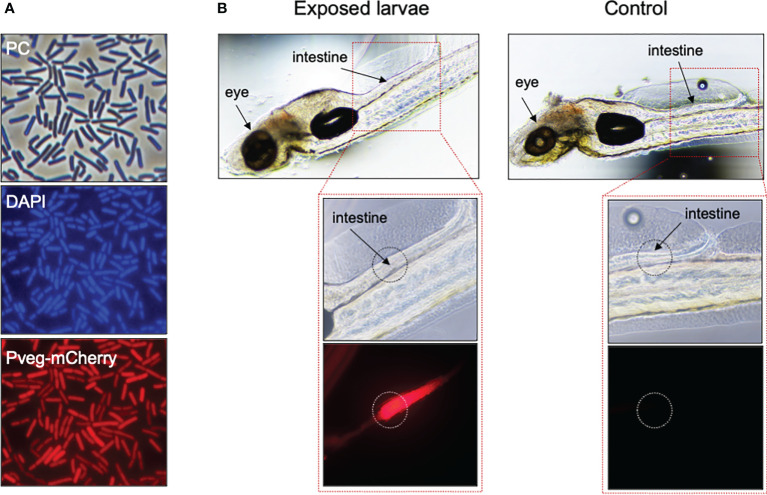
Fate of *B subtilis* cells after exposure by immersion to zebrafish larvae. **(A)** Phase contrast (PC, top panel) and fluorescence microscopy of cells of *B subtilis* derivative CRS239, bearing a P*veg-mCherry* transcriptional fusion (bottom panel), grown for 4h in DSM at 37°C and 150 rpm. Cells were labeled with DAPI, for nucleoid staining (middle panel). **(B)** Same cells of CRS239 depicted in **(A)**, after 24h exposure by immersion to 6dpf zebrafish larvae, previously treated with 75 µM PTU. Larvae exposed to CRS239 cells (Exposed) show red fluorescence at the intestinal level, while larvae that were not exposed (Control) do not.

To understand whether *B. subtilis* spores accumulated at the larvae surface or entered the larvae body, we observed by phase-contrast (PC) microscopy zebrafish larvae exposed by immersion to the same amount of spores harboring the C-terminal fusion CotY-H6-GFP in a fashion similar to that described in **Figure 2**. However, larvae autofluorescence in the green channel (not overcome by previous treatment with PTU) did not allow GFP detection (**Supplementary Figure 2**). Using an mCherry fluorescently labelled *B. subtilis* strain, it was possible to confirm that *B. subtilis* cells added to the larvae rearing water (immersion treatment) entered the zebrafish larvae body orally (probably when swallowing water upon feeding) and accumulated in the intestine **(** **Figure 3** **)**. Strain CRS239 (P*_veg_-mCherry*) was first stained with the DNA-stain DAPI and observed by PC and fluorescence microscopy (**Figure 3A**), to confirm the mCherry expression signal (bottom panel). Then, 1x10^8^ CFU mL^-1^ of CRS239 culture were added to the rearing medium of zebrafish larvae previously treated with PTU, to avoid pigment accumulation, and incubated for 24 h. When observed under fluorescence microscopy, an accumulation of red-signal could be seen in the intestine of zebrafish larvae treated with strain CRS239, but not in untreated larvae (**Figure 3B**).

### *Bacillus* spores are efficient oral vaccine delivery vehicles against Vibriosis in European seabass

**Figure 4:**
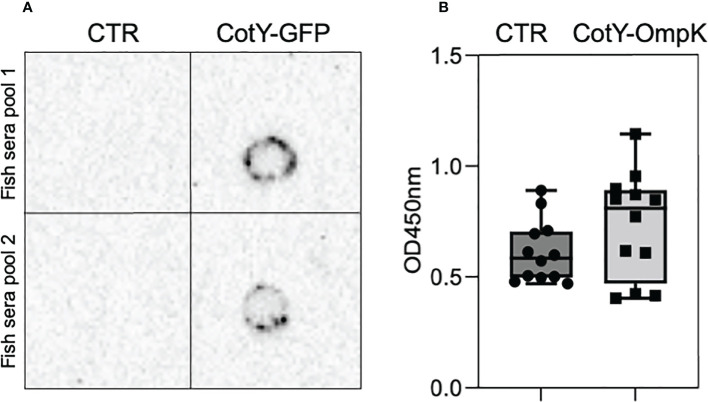
European seabass serum antibodies upon oral vaccination with OmpK and GFP carrying spores. Detection of anti-GFP **(A)** and anti-*V. anguillarum* **(B)** antibodies in the serum of orally vaccinated European seabass juveniles. Detection of anti-GFP antibodies **(A)** was done in 2 pools of 6 fish (2 fish from each tank of each experimental treatment) by Dot-Blot using purified GFP and an anti-GFP antibody. Detection of anti-*V. anguillarum* antibodies **(B)** was done by indirect ELISA in the sera of individual fish (n=12) using *V. anguillarum* cell-extracts and anti- seabass antibody. Data are represented as mean ± standard deviation (n=12).

Purified and lyophilized spores of *B. subtilis* spores carrying the C-terminal fusion of CotY to OmpK or GFP were incorporated into commercial feeds at 1x10^9^ CFU Kg^-1^ of diet and used to orally vaccinate triplicate groups of 40 European seabass juveniles for 30 days. At the end of the trial, serum of fish fed the GFP-carrying spores diet was used to determine anti-GFP antibody production by Dot-Blot with a purified GFP and a GFP-specific antibody (**Figure 4A**). Simultaneously, serum of fish vaccinated with OmpK-carrying spores diet was used to indirectly determine anti-OmpK antibody production by ELISA, using *V. anguillarum* cell-extracts and an anti-seabass antibody (**Figure 4B**). The serum of fish fed the GFP-carrying spores diet reacted with the purified GFP, while serum from fish fed the un-supplemented diet (CTR) did not react (**Figure 4A**), an indication that fish fed the GFP-carrying spores diet did produce GFP-specific antibodies. ELISA analysis of serum of fish vaccinated with OmpK-carrying spores diet revealed a higher titer of anti-*V. anguillarum* antibodies, although not significantly different from unvaccinated fish, whose sera also reacted in the ELISA analysis (**Figure 4B**).

**Figure 5:**
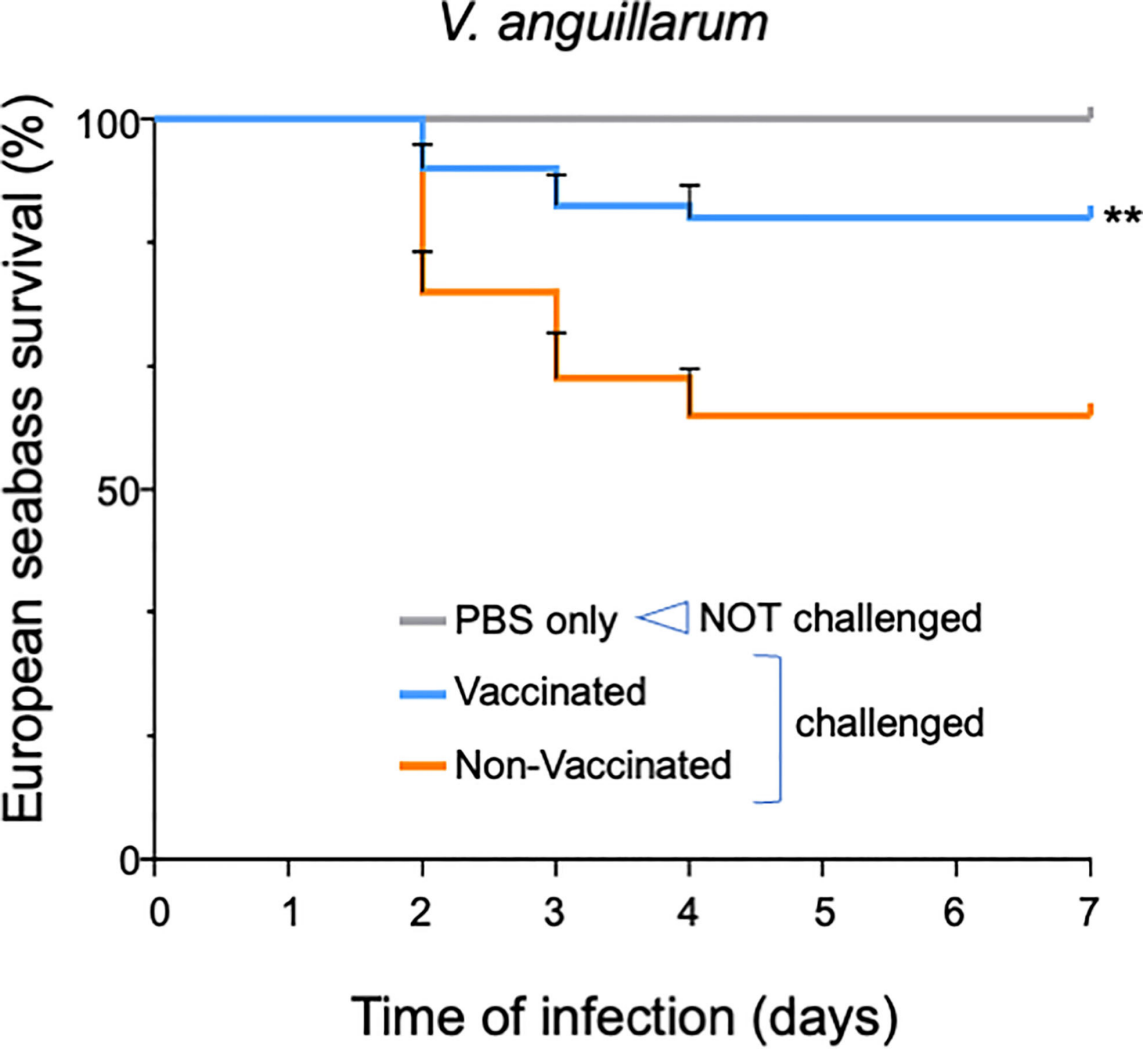
European seabass survival to *V. anguillarum* infection after oral vaccination with OmpK carrying spores. Survival Kaplan-Meyer survival curves of European seabass juveniles upon challenge with *V. anguillarum* after being orally vaccinated for 1 month with feed containing spores bearing the OmpK antigen fused to CotY (CRS220, CotY-H6-OmpK). Non-vaccinated fish and non-challenged fish (PBS only) were used as positive and negative control groups, respectively. Fish were monitored for 15 days. Data are represented as mean ± standard deviation of three independent biological experiments. Significant differences are highlighted by asterisks ** (*p*<0.001).

OmpK-vaccinated and non-vaccinated fish were then submitted to a bacterial challenge by intra-peritoneal (i.p.) injection with 100 µl of 1x10^7^ CFU mL^-1^ *V. anguillarum* (challenged fish) or with 100 µl of PBS only (non-challenged fish). Compared to non-challenged fish, in which no mortality occurred, upon *V. anguillarum* challenge non-vaccinated fish experienced a 40% mortality while in vaccinated fish the mortality was significantly reduced to 13% (*p*<0.05) (**Figure 5**).

## Discussion

*B. subtilis* is a generally regarded as safe (GRAS) spore-forming bacterium, commonly used in biotechnological applications (51, 52). *B. subtilis* spores are promising delivery vehicles of different molecules through surface display technology (15, 17, 53, 54). By expressing different target proteins on their surface, *B. subtilis* spores have been successfully used as bioremediation products (55), biopesticides (56), drug delivery systems (57), vaccine vehicles (18, 19, 58–61), industrial biocatalysts (62) and animal probiotics (53), among others, as recently reviewed (15, 17, 53, 54).

The extreme resistance of *B. subtilis* spores, which guarantees passage through the harsh gut environment without losing characteristics (63), is the basis for their oral applications as probiotics or as delivery vehicles (20, 24, 26–29, 31, 32, 60, 64–68). Spores’ direct incorporation into animal feed, circumventing further protection processes, such as encapsulation, together with their simple production and long shelf-life without needing refrigeration (51, 69), are also attractive characteristics from the industrial point of view (70). Adding to this “needle-free” and “refrigeration-free” potential, *B. subtilis* spores adjuvant properties (18, 19, 71–73) and contribution to GALT (gut associated lymphoid tissue) development (67, 74–76), increases their potential as oral delivery systems of antigens. Previous reports have shown the application of *B. subtilis* spores as oral (or mucosal) vaccines in different animals, including aquatic animals (20, 24, 26–29, 31, 32, 60, 64–68). Oral vaccination is of utmost importance in aquaculture, where individual handling of fish for injection vaccination, provokes stress-related mortalities (6, 11, 77–79).

The success of oral (or mucosal) spore-based vaccination will largely depend on the success of the target protein display, which is determined by the correct combination of the *B. subtilis* protein used as anchor with the pathogen protein used as the target antigen (80, 81). The choice of a correct fusion and anchor partner is thus critical. Since its first description, using CotB as an anchor protein to display the tetanus toxin (TTFC) on the surface of *B. subtilis* spores (80), different spore-coat proteins (e.g. CotC, CotG, OxdD, CotZ) have been used as anchors for spore surface display [revised in (17)]. In this work, we selected CotY as the anchor for spore surface display, based on the studies from Bartels et al. (36) that found it to be the best anchor protein for both C- and N-terminal fusions. CotY is located on the spore crust, the most superficial layer, which allows the antigen to be exposed extracellularly and accessible to the immune system (36, 82). Transcription of the fusions was under the control of the P*_cotYZ_* promoter, which is the strongest intrinsically activated crust promoter (36, 82).

Using the crust CotY protein as a carrier, we were able to display two target proteins at the surface of *B. subtilis* 168 spores, the green fluorescence protein, GFP, which we used as a control for display success, and the outer membrane protein K, OmpK, which is shared among *Vibrio* spp. (83), as our target antigen against vibriosis. Vaccine development focused on antigens that are common among the target pathogen, might enhance the chances of obtaining more effective and versatile vaccines. Outer membrane proteins (OMP), such as OmpK, are considered common immunogenic proteins among *Vibrio* spp. (83, 84). Although other antigens have been pointed out as vaccine candidates, like TolC, an outer membrane protein from *V. harveyi* (85) and DNAj (86), several experimental vaccines using OmpK were described as efficient. For instance, vaccination of Orange-spotted groupers with a recombinant OmpK induced a higher tolerance to *Vibrio* infections (83); a subunit vaccine involving OmpK and OmpU fusion resulted in a protective effect against *V. harveyi* infection and showed potential against other species (87); *E. coli* based recombinant vaccines containing OmpK were efficient against challenge with multiple strains of *V. harveyi*, *V. alginolyticus*, and *V. parahaemolyticus (* 86 *).* Nonetheless, studies regarding either the immunogenicity of OmpK or the cross-protection effect of recombinant OmpK vaccines are not consistent. For instance, in a study using recombinant OmpK, recombinant GAPDH, and a fusion of both antigens as possible vaccines, although antibody titration from vaccinated groups was significantly different from the control group, the protective effect upon challenge was better using the fusion of both antigens (88). In another study (89), serum from mice immunized with recombinant OmpK from *V. parahaemolyticus* only reacted with homologous *V. parahaemolyticus*, indicating that recombinant OmpK may not be the ideal target antigen for the development of a polyvalent vaccine.

To maximize chances of success in protein display at the surface of *B. subtilis* spores, GFP and OmpK were both C- and N-terminally fused to CotY. Although sequencing of the target genomic region indicated that all fusions were correctly integrated into *B. subtilis* chromosome *via* a double cross-over event at the *amyE* locus, we were not able to detect by western blot the N-terminal versions of both OmpK (H6-OmpK-CotY) or GFP (H6-GFP-CotY) fused with CotY. On the contrary, C- terminal recombinant fusions of OmpK (CotY-H6-OmpK) or GFP (CotY-H6-GFP) to CotY were successfully detected by western blot. GFP display at the spores’ surface was further confirmed by fluorescence microscopy in both derivatives, and the C-terminal version CotY-H6-GFP exhibited a higher fluorescence signal than the N-terminal H6-GFP-CotY. A possible explanation for the inability to detect the N-terminal fusions by western blotting is the strong and tight interaction network formed by cross-linked protein complexes that are present at the spore outermost layers structure (43, 90–92). CotY appears to interact with most of the spore crust proteins, including itself. Indeed, the strongest functional complexes involving this protein have arisen from self-interactions (82, 90–94). Moreover, considerably strong bonds between CotY-CotZ and CotY-CotV have been frequently observed (90–92). These protein complexes may be resistant to the degree of denaturation performed, and thus unlikely to run in the polyacrylamide gel. On the other hand, the 6His-tag epitope (used in our study to detect the fusions by western blot) may be hidden in the complex, preventing antibody interaction. Although Bartels et al. (36) did not observe major differences in display efficiency whether using C- or N-terminally fused proteins to CotY, our observations suggest that N-terminal fused proteins are less efficiently displayed at the surface of *B. subtilis* spores when using CotY sporovector (36). Nevertheless, although spores are compact and tight structures, carefully organized to achieve optimal resistance properties (90–92), the display of heterologous GFP or OmpK did not affect sporulation and resistance to heat and lysozyme of the recombinant strains. These are essential characteristics for their application as oral antigen delivery vehicles, as spores must resist passage through the harsh environment of the fish gastrointestinal tract.

The recombinant strains were then tested as potential delivery vehicles for fish, using as a first approach zebrafish larvae, a model organism recurrently used for immunological research (78, 95–97). Zebrafish’s practical advantages, such as high breeding rate, rapid development, and low maintenance costs, make these animals ideal for high throughput tests (97, 98). A preliminary toxicity test indicated that we could expose the zebrafish larvae to 1x10^8^ CFU mL^-1^ of a suspension of *B. subtilis* 168 spores (data not shown) without any visible harmful effect.

As an attempt to evaluate vaccine potential against vibriosis, zebrafish larvae were then exposed to a suspension of purified spores from strains bearing the CotY-OmpK fusions (CRS220 and CRS221) three days before being infected with *V. anguillarum* and *V. parahaemolyticus*. Vaccination with spores displaying the C-terminal fusion CotY-H6-OmpK significantly increased the survival of larvae challenged with *V. anguillarum* (by 85%) and *V. parahaemolyticus* (by 40%). Further, survival of unvaccinated larvae or larvae “vaccinated” with spores of the WT parental strain (not carrying the CotY-OmpK) was not significantly different from each other, indicating that it was the antigen and not the spore itself responsible for the increased protection observed. Nevertheless, an adjuvant contribution of the spores cannot be ruled out, as it has been previously described both in mammals and in fish (45, 72, 73, 99).

Although provided by immersion, *B. subtilis* accumulated in the zebrafish larvae intestine, likely entering when larvae swallowed water upon feeding, which indicates that the observed protection might be happening at the intestinal level. In humans, oral immunization stimulates an immune response that is effective against both mucosal and systemic infections (100). In fish, oral immunization is thought to preferably induce a mucosal immune response accompanied by secretion of immunoglobulins IgM, IgD, and IgT/Z (13, 14). The fish intestinal mucosa is one of the main routes for pathogens translocation into the body to cause disease; thus, inducing a “frontline” mucosal immunity (via bath or oral vaccination) is of utmost importance. A previous report using bath vaccination of zebrafish with an attenuated *V. anguillarum* vaccine also observed an accumulation of bacteria at the intestinal level, while inducing a gut mucosal immune response (96). Although we did not address the immunological mechanisms associated with the increased zebrafish larvae survival, a similar mucosal immune response might likely have been induced at the intestinal level. Similarly, previous works have described that both *B. subtilis* spores and *B. subtilis* cells carrying antigens can induce systemic and mucosal immune responses in mammals, including in distal mucosal tissues (70). In fish, spore-based vaccines were described to increase *Clonorchis sinensisis* antigen-specific IgMs in grass carps sera, intestinal and skin mucus (30, 31); trigger both innate and adaptive humoral and cellular immune responses and resistance to reovirus infection (26–28); up-regulate transcription of immune-related genes while increasing survival upon infection with red-spotted nervous necrosis virus in grouper (68) or with *Streptococcus agalactiae* in tilapia (32).

To further evaluate the potential of engineered *B. subtilis* strains as oral vaccines for fish, both GFP and OmpK containing spores were mixed with commercial feed for European seabass and used to orally vaccinate juveniles for one month. European seabass is both an economically important species in aquaculture and a marine model species in immunology (101). By the end of the experiment, anti-GFP antibodies were detected in the serum of fish fed the GFP-carrying spores supplemented diet (and not in fish fed the placebo diet) using a purified GFP and a GFP-specific antibody. Direct detection of anti-OmpK antibodies in the serum of fish vaccinated with OmpK-carrying spores supplemented diet was not possible due to the lack of a specific anti-OmpK antibody. Instead, an indirect approach using immobilized *V. anguillarum* cell extracts and an anti-seabass antibody was used to infer the immunogenicity of the OmpK-carrying spores. Although a reaction signal was obtained in both vaccinated and non-vaccinated fish, the levels were higher in the vaccinated group. In opposition to GFP, which fish are not used to contact, *Vibrio* spp. are spread within the aquatic environment and within the fish gut, and this might explain the detection of anti-*V. anguillarum* antibodies in the sera of non-vaccinated fish. Further, our spore-based vaccine contained OmpK as an immunogenic protein, but we used entire *V. anguillarum* extracts for detection which may also have contributed to the sub-estimation of the response. Another explanation is the occurrence of oral tolerance to our spore-based vaccine, due to prolonged/repetitive exposure to the OmpK antigen. Oral tolerance is a phenomenon of systemic immunity suppression (with the generation of regulatory T-cells) that occurs to avoid unnecessary immune responses against normal gut commensals and certain food-derived antigens while maintaining an active reaction against enteric pathogens (14). Previous studies in fish described the occurrence of oral tolerances, inferred by gradual down-regulation of immunity genes expression or suppression of antibody production (14). Such mechanism is however unlikely to have occurred in our study since OmpK-vaccinated European seabass juveniles showed significantly lower mortality when challenged with *V. anguillarum* than did non-vaccinated fish. The mortality of orally vaccinated European seabass was reduced from 40% to 13%. The previously reported adjuvant properties of *B. subtilis* spores (45, 72, 73, 99) together with the immunomodulatory capacity of their metabolites (40, 102) might have contributed to the protection observed (14).

Altogether our results indicate that *B. subtilis* spores displaying the immunogenic protein OmpK at their surface protect two fish species (zebrafish and European seabass), from 2 different environments (freshwater *vs* saltwater), and at 2 different developmental stages (larvae *vs* juveniles) against vibriosis etiological agent, *Vibrio anguillarum*, and are thus promising vaccine vehicles (SporoVaccines) against vibriosis in fish.

## Data availability statement

The raw data supporting the conclusions of this article will be made available by the authors, without undue reservation.

## Ethics statement

Animal experiments were approved by the Animal Welfare Committee of the Interdisciplinary Centre of Marine and Environmental Research (CIIMAR) and carried out in a registered installation (N16091.UDER) and were performed by trained scientists (following FELASA category C recommendations) in full compliance with national rules and following the European Directive 2010/63/EU of the European Parliament and the European Union Council on the protection of animals used for scientific purposes.

## Author contributions

Conceived and designed the experiments: GG, RS, FC, PD-R, ACo, CS. Fish maintenance and sampling: GG, RS, FC, MM, ACa, ACo, CS. Performed the experiments and analyzed the data: GG, RS, NP, MC, LB, MS, CS. Critically evaluated all the data and edited manuscript: GG, RS, BC, AO-T, PD-R, ACo, CS. Wrote the paper: GG, CS. All authors contributed to the article and approved the submitted version.

## Funding

This work was financed by Fundo Europeu de Desenvolvimento Regional (FEDER) funds through the COMPETE 2020 Operacional Programme for Competitiveness and Internationalisation (POCI), Portugal 2020, and by Portuguese funds through Fundação para a Ciência e a Tecnologia (FCT)/Ministério da Ciência, Tecnologia e Ensino Superior in the framework of the project PTDC/CVT-CVT/2477/2021. FCT is also greatly acknowledged for the PhD fellowships 2021.07724.BD (GG) and SFRH/BD/131069/2017 (RS) and for the scientific employment contract of ACo and CS. This research was also partially supported by CIIMAR Strategic Funding UIDB/04423/2020 and UIDP/04423/2020 through national funds provided by FCT and FEDER, in the framework of the programme PT2020.

## Conflict of interest

The authors declare that the research was conducted in the absence of any commercial or financial relationships that could be construed as a potential conflict of interest.

## Publisher’s note

All claims expressed in this article are solely those of the authors and do not necessarily represent those of their affiliated organizations, or those of the publisher, the editors and the reviewers. Any product that may be evaluated in this article, or claim that may be made by its manufacturer, is not guaranteed or endorsed by the publisher.
