# Supplementary material for: Oral vaccination of fish against vibriosis using spore-display technology
Source: Front Immunol. 2022 Oct 13;13:1012301. doi: 10.3389/fimmu.2022.1012301 (PMC9608137; doi:10.3389/fimmu.2022.1012301)
Supplement: Supplementary file 1 [file DataSheet_1.pdf]

Supplementary Material for

Oral vaccination of fish against vibriosis using spore-display technology

Gabriela Gonçalves [1,2,#], Rafaela Araújo [1,2,#], Filipe Coutinho [2], Neide Pedrosa [2], Maria Curado [3], Marina Machado [3], Benjamin Costas [2,4], Lourenço Bonneville [5], Mónica Serrano [5], António Paulo Carvalho [1,2], Patricia Díaz-Rosales [6], Aires Oliva-Teles [1,2], Ana Couto [1,1], Cláudia R. Serra [1,2,*]

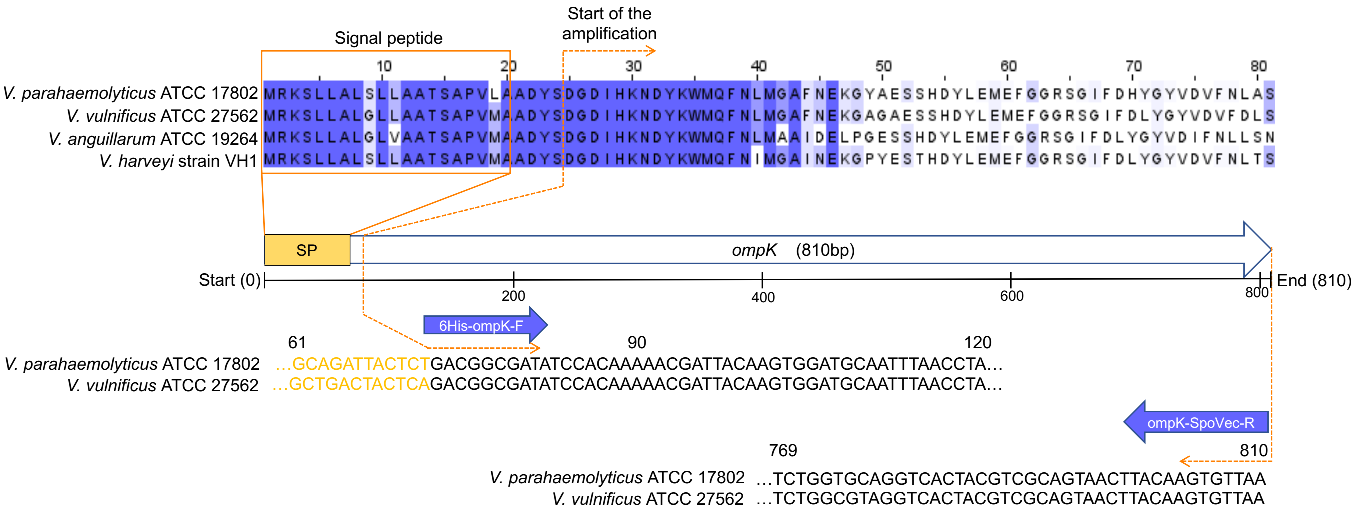

Supplementary Figure 1. Schematic representation of the strategy used to amplify the OmpK antigen. The amino acid sequences of the OmpK protein from *V. parahaemolyticus* ATCC 17802, *V. vulnificus* ATCC 27562, *V. anguillarum* ATCC 19264 and *V. harveyi* strain VH1 were aligned using Jalview 2.11.17 software (only the first 80 aa are presented), with the signal peptide (SP) corresponding to the first 20 aa, indicated. The amplification of the *ompK* sequence started at nucleotide 73 (amino-acid 25), that corresponds to a conserved region among all strains analysed and ended at the last nucleotide of the gene.

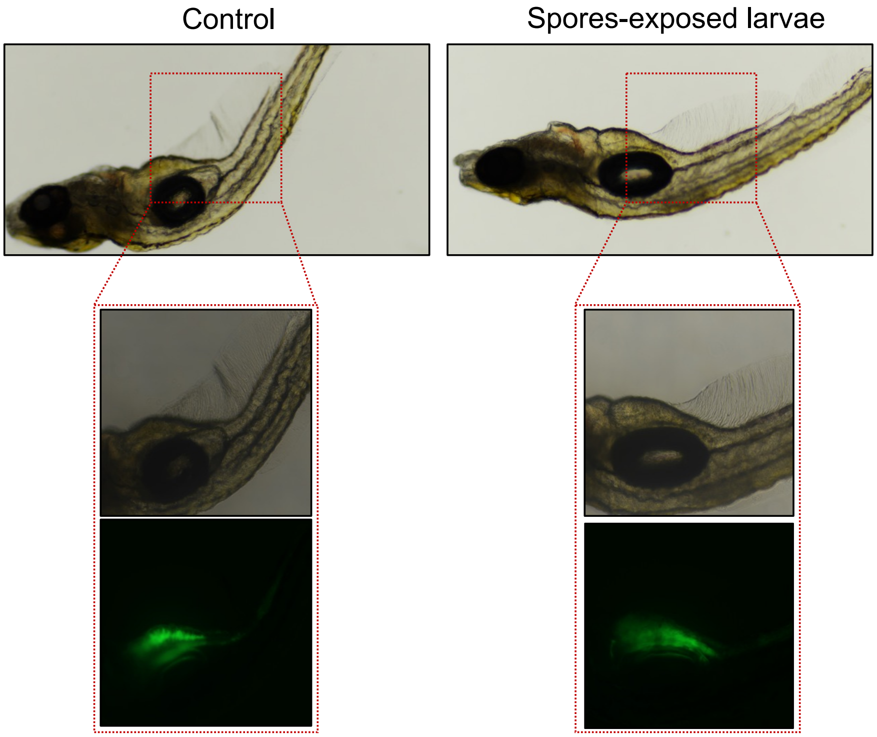

Supplementary Figure 2. Zebrafish larvae (6 dpf), previously treated with 75 µM PTU, were exposed by immersion to a spores suspension from strain CRS218 (Spores-exposed larvae). Both spores-exposed and unexposed larvae (Control) showed green fluorescence at the intestinal level.

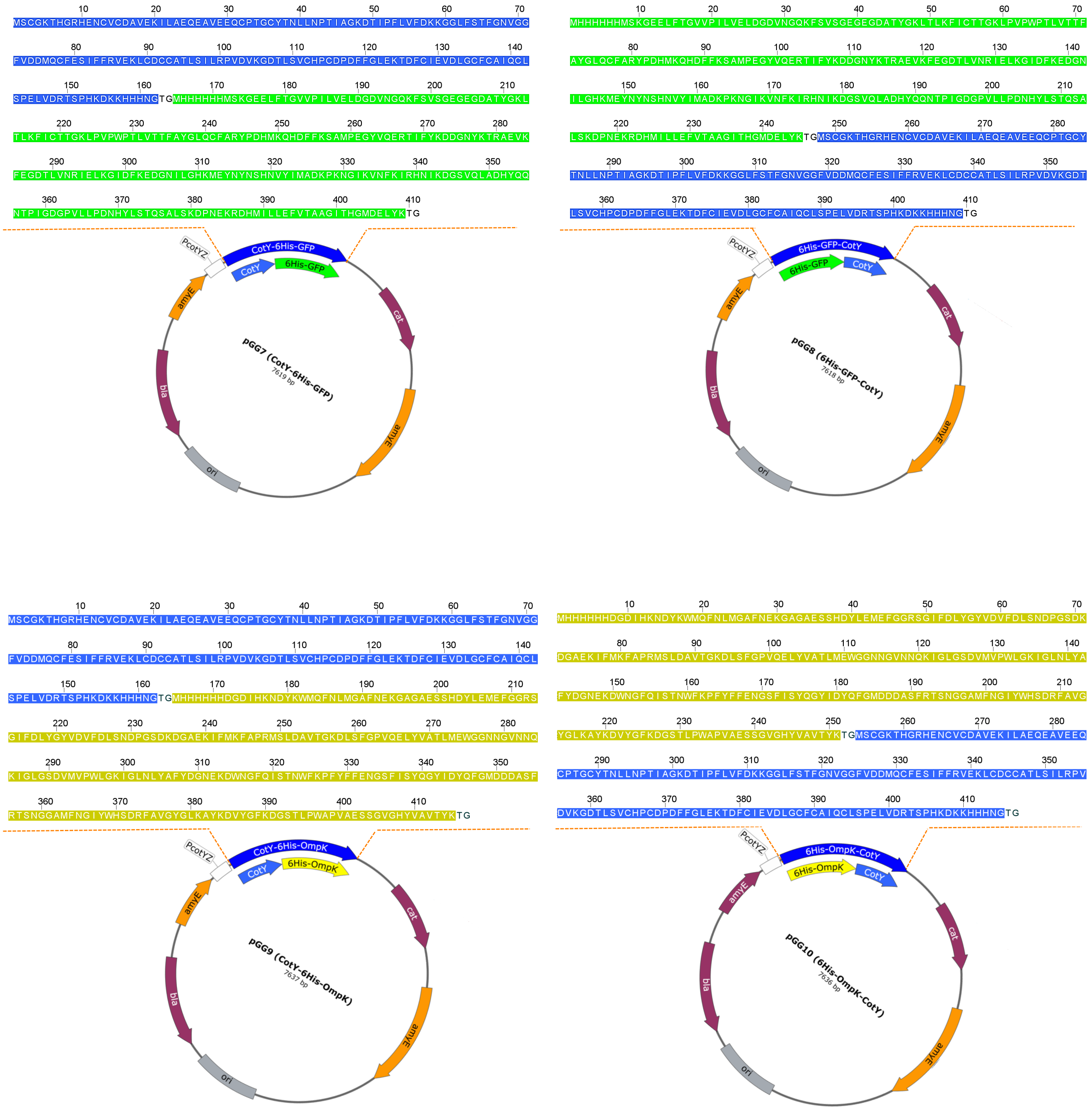

Supplementary Figure 3. Representative plasmid maps of pGG7 (CotY-6His-GFP), pGG8 (6His-GFP-CotY), pGG9 (CotY-6His-OmpK) and pGG10 (6His-OmpK-CotY). The amino acid sequence of each fusion is represented. Plasmid maps and amino acids sequence were constructed with SnapGene software (version 6.1) and Jalview software (2.11.2.4)
